# Urticaria in Pregnancy and Lactation

**DOI:** 10.3389/falgy.2022.892673

**Published:** 2022-07-07

**Authors:** Emek Kocatürk, Indrashis Podder, Ana C. Zenclussen, Alicja Kasperska Zajac, Daniel Elieh-Ali-Komi, Martin K. Church, Marcus Maurer

**Affiliations:** ^1^Department of Dermatology, Koç University School of Medicine, Istanbul, Turkey; ^2^Department of Dermatology, Venereology and Leprosy, College of Medicine and Sagore Dutta Hospital, Kolkata, India; ^3^Department of Environmental Immunology, Helmholtz Centre for Environmental Research (UFZ) and Saxonian Incubator for Clinical Translation (SIKT), University of Leipzig, Leipzig, Germany; ^4^European Center for Diagnosis and Treatment of Urticaria/Angioedema (GA2LEN UCARE /ACARE Network), Zabrze, Poland; ^5^Department of Clinical Allergology, Urticaria Center of Medical University of Silesia, Katowice, Poland; ^6^Institute of Allergology, Charité – Universitätsmedizin Berlin, Corporate Member of Freie Universität Berlin and Humboldt-Universität zu Berlin, Berlin, Germany; ^7^Fraunhofer Institute for Translational Medicine and Pharmacology ITMP, Allergology and Immunology, Berlin, Germany

**Keywords:** urticaria, pregnancy, lactation, treatment, autoimmunity, immunological changes, mast cells, hormones

## Abstract

Chronic urticaria (CU) is a mast cell-driven chronic inflammatory disease with a female predominance. Since CU affects mostly females in reproductive age, pregnancy is an important aspect to consider in the context of this disease. Sex hormones affect mast cell (MC) biology, and the hormonal changes that come with pregnancy can modulate the course of chronic inflammatory conditions, and they often do. Also, pregnancy-associated changes in the immune system, including local adaptation of innate and adaptive immune responses and skewing of adaptive immunity toward a Th2/Treg profile have been linked to changes in the course of inflammatory diseases. As of now, little is known about the effects of pregnancy on CU and the outcomes of pregnancy in CU patients. Also, there are no real-life studies to show the safety of urticaria medications during pregnancy. The recent PREG-CU study provided the first insights on this and showed that CU improves during pregnancy in half of the patients, whereas it worsens in one-third; and two of five CU patients experience flare-ups of their CU during pregnancy. The international EAACI/GA^2^LEN/EuroGuiDerm/APAAACI guideline for urticaria recommends adopting the same management strategy in pregnant and lactating CU patients; starting treatment with standard doses of second-generation (non-sedative) H1 antihistamines, to increase the dose up to 4-folds in case of no response, and to add omalizumab in antihistamine-refractory patients; but also emphasizes the lack of evidence-based information on the safety and efficacy of urticaria treatments during pregnancy. The PREG-CU study assessed treatments and their outcomes during pregnancy. Here, we review the reported effects of sex hormones and pregnancy-specific immunological changes on urticaria, we discuss the impact of pregnancy on urticaria, and we provide information and guidance on the management of urticaria during pregnancy and lactation.

## Introduction

Chronic urticaria (CU) is a chronic inflammatory disorder, which presents with the sudden and unpredictable appearance of wheals, angioedema, or both for longer than 6 weeks (1). CU is a female dominant disease with a higher diagnosed incidence (0.18 vs. 0.11%) and prevalence (0.62 vs. 0.37%) of females vs. males (2). Recently a meta-analysis showed that chronic spontaneous urticaria (CSU) has a point prevalence of 1.3 and 0.8% in women vs. men and chronic inducible urticaria (CIndU) shows a female: male ratio of 2:1 to 3:1 (3). From the results of the recent AWARE study, which focused on worldwide management patterns of antihistamine-refractory CU, it is also clear that rates of female CU are higher than those of males, i.e., 72% for CSU and 69.8% for CIndU (4). CU is not only more common in females but also more severe, with higher rates of high disease activity, angioedema, poor prognosis, refractoriness to treatment, and longer disease course (5–9). The lack of female predominance in children younger than 15 years (3) suggests a disease-modifying role for female hormones in CU. Female sex hormones can influence inflammatory diseases including autoimmune conditions in many different aspects. They are considered risk factors of disease onset and are held to contribute to the activity and progression of autoimmune diseases (10, 11).

**Figure 1:**
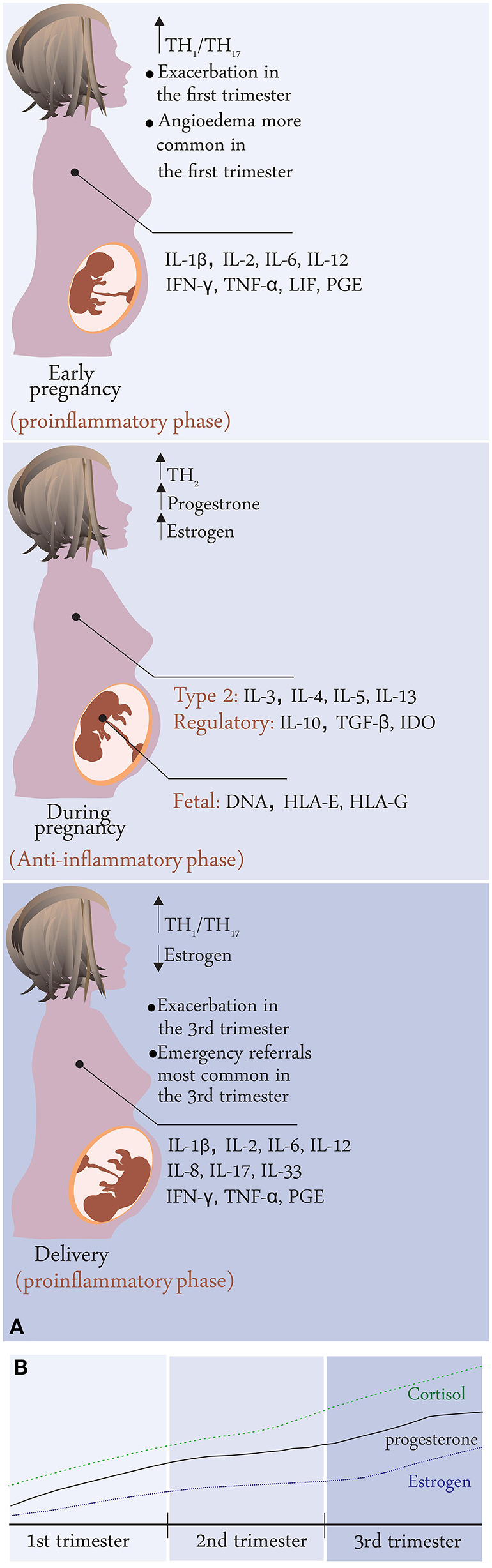
**(A)** Disease activity changes during pregnancy in CU patients; summary of results from the PREG-CU study with possible mechanisms related to immunological changes in pregnancy. **(B)** Change in hormone levels during pregnancy.

From the clinical experience, change in hormone levels, for example across menstrual cycle or during pregnancy, with the onset of menopause, or as a result of using hormonal contraceptives or hormone replacement therapy, some changes in disease activity might be observed. Further, because CU affects mostly women in reproductive age, it is important to understand the consequences of hormonal changes within the menstrual cycle, because of hormonal contraception and during pregnancy for CU disease course and severity. Robust data on this is scarce, but a recent multicenter study revealed that CU tends to improve during pregnancy in half of the patients and worsen in one third of them (Figure 1). Worsening of urticaria was associated with having a mild disease before pregnancy and not being on treatment before pregnancy (12). These findings stress the importance of proper clinical and laboratory diagnosis as well as treatment of CU for patients willing to get pregnant and also a personalized follow-up during pregnancy. Therefore, optimal management of urticaria during pregnancy is vital to ensure the best outcome for the mother and the baby, however, medications' potential risks must be balanced against the consequences of untreated disease.

In this review, we are going to focus on the effect of sex hormones on urticaria, disease activity changes during pregnancy, and management of urticaria during pregnancy and lactation.

## Hormonal and Immunological Changes During Pregnancy

A number of important hormonal changes that modulate the immunological milieu take place during pregnancy. Some of these changes may influence CU during this period as highlighted below.

### Hormonal Changes

In addition to the emergence of the pregnancy-specific hormone human chorionic gonadotrophin (hCG), several other hormones are upregulated during pregnancy such as progesterone (P4), estrogens, cortisol, prolactin, leptin, vitamin D, and alpha-fetoprotein (AFP).

HCG is a placental glycoprotein hormone, that appears first during pregnancy and serves as pregnancy confirmation, peaks during the 9th−12th week of pregnancy, followed by a gradual decline until delivery, even though hCG levels during pregnancy remain high. Its major function is to maintain P4 synthesis by the corpus luteum (13). HCG promotes maternal immune tolerance and helps to ensure fetal survival (14). The hormone is able to convert naïve T cells into regulatory T cells (Treg) (15); can modulate dendritic cells (DCs) into tolerogenic antigen-presenting cells (APCs) (16, 17) and stimulate IL-10 production by B cells (18). HCG application was shown to *in vivo* boost the number of Treg cells and prevent abortion in mice (14) and is also injected intrauterine in IVF protocols for women with a history or implantation failure (19).

Progesterone (P4), is a member of the steroid hormone family and plays a crucial role in maintaining pregnancy, in addition to HCG (14). During the initial stages, it is secreted by the corpus luteum and, later on, by the placenta. P4 modulates the immune system *via* intracellular P4 receptors (PR) expressed by epithelial cells, eosinophils, macrophages, lymphocytes MCs, and DCs (20), and by the upregulation of progesterone-induced blocking factor (PIBF) and glycodelin A (a cell-surface glycoprotein expressed in endometrium/decidua, amniotic fluid, and maternal serum, with immunosuppressive properties) (14). P4 contributes to gestational tolerance by suppressing innate immunity *via* different mechanisms such as blocking the cytolytic action of NK cells, inducing tolerogenic DCs, and promoting Th2 polarization by preferential apoptosis of Th1 subset and increasing Th2 cytokine production (20, 21). Furthermore, P4 stimulates Treg cells by inducing Fork Head Box Protein 3 (FoxP3) expression in naïve T-cells at the feto-maternal interface, in murine pregnancies (21). P4 plays a crucial role in maintaining gestational tolerance by suppressing innate immunity and promoting Th2 polarization (20). The maternal P4 level continues to rise until about 10–12 weeks of gestation (corpus luteum), then returns to its baseline level and again starts to rise around the 32nd week of pregnancy (2nd peak-secreted by the placenta) to be maintained until conception (22). P4 levels drop during lactation (23). Occasionally, CSU-like cutaneous eruption has been reported due to excess serum P4, called progesterone hypersensitivity. The reasons for increased serum P4 include pregnancy or exposure to exogenous progesterone or increased level during the menstrual cycle (luteal phase; 3–10 days before the onset of menstruation) (24).

Estrogens, also belonging to the steroid hormone family, are of three major types, estrone (E1), estradiol (E2), and estriol (E3). Among them, E2 constitutes the major fraction in reproductive females (both pregnant and non-pregnant) and accounts for most of the classic estrogenic-induced effects. In contrast, E3 is exclusively secreted in pregnant females, by the fetoplacental unit, and comprises almost 90% of the pregnancy estrogen (20). During pregnancy, estrogen levels rise steadily until delivery due to placental secretion. Estrogens act *via* estrogen receptors (ERs) expressed by B and T lymphocytes, macrophages, and DCs and affect both innate and acquired immunity (14). It is generally accepted that estrogens play a role in the development of adaptive immunity such that low levels of estrogen promote pathogenic Th1/Th17 pathway while high levels (as during pregnancy) promote Th2/Treg responses. E3 downregulates innate immunity by programming DCs to become tolerogenic and anti-inflammatory (23). Many researchers have depicted the harmful role of exogenous estrogen mimickers, called endocrine-disrupting chemicals (EDCs), which act by disrupting the endocrine milieu. These substances are present in several daily use products such as plastic water bottles or food containers, which may be systemically absorbed by ingestion. Recently, the negative impact of EDCs, particularly Bisphenol A and phthalates, is being recognized on human pregnancy and fetal development by interfering with the developing embryonic epigenome (24). Rarely, premenstrual urticarial eruption has been reported in women with estrogen hypersensitivity. In such cases, removal of the exogenous estrogen results in remission e.g., discontinuation of estrogen-containing oral contraceptives or use of estrogen antagonists (leuprolide or tamoxifen) (25).

Cortisol, synthesized in the adrenal cortex and released into circulation after various physical and psychological stimuli; has strong anti-inflammatory effects. During pregnancy, maternal cortisol levels rise continuously to facilitate fetal development, followed by an abrupt drop post-partum (26). Cortisol exerts its anti-inflammatory effect by multiple pathways such as reducing the circulating levels of pro-inflammatory cytokines such as IL-2, IL-3, IL-6, IFN-γ, and TNF-α, activating tolerogenic Treg cells by enhancing the expression of high-affinity IL-2 receptor (CD25), inducing the apoptosis of T cells, and reducing the number of B cells in spleen and lymph nodes, thereby reducing IgG production (23). These effects may explain, in part, the improvement of some immunological disorders during pregnancy.

Prolactin, a polypeptide hormone, is largely produced by the lactotrophic cells of the pituitary gland and several extra-pituitary sources like mammary epithelium, ovaries, and placenta, under the influence of dopamine. Prolactin levels increase slightly during gestation with exponential rise during delivery and lactation, contrasting the abrupt reduction of sex hormones (E2, E3, and P4) post-delivery (27). Prolactin stimulates the immune system and causes aberrant activation by aiding the maturation of naïve Th0 cells to effector CD4 and CD8 cells, impairing the clonal deletion of auto-reactive B cells, and reducing the threshold for activating anergic B cells. Thus, hyperprolactinemia has been associated with several autoimmune disorders and might explain disease flare or relapse during breastfeeding (28). The effects of prolactin on CU during pregnancy remain largely unexplored, but Sabry et al. (29) reported significantly higher serum prolactin levels in a subset of CU patients (positive autologous serum skin test, ASST) and its association with disease severity. In contrast, Soliman et al. (30) did not find any relationship between serum prolactin levels and urticaria activity.

Leptin, secreted by adipocytes, primarily regulates energy metabolism, but its impact on the immune system is increasingly being recognized. Leptin promotes inflammatory responses by activating the JAK-STAT, PI3K, and MAPK pathways as its receptor mimics the IL-6 receptor (31). A recent review has highlighted the cross-talk between mast cells and adipocytes in certain situations like obesity, where adipose tissue-resident MCs release pro-inflammatory cytokines like TNF-α, under the influence of leptin, and worsen the inflammatory state (32). During pregnancy, leptin levels rise to counter the hypermetabolic state and modulate the feto-maternal immune system. The recent findings that the placenta is a relevant source of leptin and its trophoblastic effects further strengthen this view (33). Several authors have reported higher serum levels of leptin in patients with CU (34, 35), but, as of yet, pregnant CU patients have not been studied.

Vitamin D, a steroid hormone, plays an important role in modulating the immune system during pregnancy. The placenta is one of the major sites of extra-renal vitamin D synthesis and produces considerable amounts during pregnancy. Vitamin D promotes antibacterial innate immune responses and suppresses inflammatory adaptive immunity *via* negative effects on NK cells, T, and B cells (36). The effect on T-cells include a shift from Th1 to Th2 phenotype, *in vitro* suppression of Th17 axis and IL-17 secretion, and inducing the conversion of naïve T-cells into tolerogenic Treg cells, while antibody production by B-cells is suppressed by inhibiting the differentiation of plasma cells and memory cells (23). Furthermore, placental vitamin D contributes to the development of localized fetal-maternal immune tolerance (37). Vitamin D deficiency may negatively affect several immune-mediated disorders, such as psoriasis, type 1 diabetes, multiple sclerosis, rheumatoid arthritis, tuberculosis, sepsis, and systemic lupus erythematosus (23, 38). A recent systematic review concluded that adult patients with CU are at a higher risk of developing Vitamin D deficiency, and its supplementation may provide therapeutic benefit in this subset (39).

Alpha-fetoprotein (AFP) is another pregnancy-specific glycoprotein hormone secreted by the yolk sac and fetal liver. This hormone peaks between weeks 12 and 16 of pregnancy, and gradually declines thereafter. AFP may have immune regulatory effects, but conclusive evidence is lacking (40).

### Immunological Changes

The human immune system is designed to recognize and eradicate possibly harmful foreign, i.e., non-self antigens. During pregnancy, paternal antigens that are expressed by the fetus are recognized as foreign, but the maternal immune system protects the fetus through several immunological changes briefly discussed here.

#### Changes in Innate Immunity

Innate immunity refers to the inborn, non-specific, immediate host defense against any antigen, which does not require a previous sensitization. During pregnancy, the innate immune system and its effector cells change considerably, and this adaptation is important rather locally, primarily aimed at uterine vascular remodeling for fetal development. Among the various components of innate immunity, uterine NK cells (uNK) constitute the most important population. The important changes pertaining to innate immunity are briefly discussed below.

##### Dendritic Cells

Dendritic cells (DCs) are vital APCs and act as a conduit between innate and adaptive immunity. During pregnancy, P4, E2, and hCG stimulate most of the uterine/decidual DCs to become tolerogenic and secrete the anti-inflammatory cytokine IL-10, thus creating a favorable local environment for the growing fetus (14). This is supported by a study by Segerer et al. (41) and Wan et al. (42), who reported significant up-regulation of IL-10 secretion by human DCs when stimulated *in vitro* by pregnancy hormones. Additionally, sex hormone-primed decidual DCs demonstrate impaired up-regulation of MHC-II and other co-stimulatory molecules, thereby reducing their ability to secrete proinflammatory cytokines (43). Interestingly, these effects are restricted to uterine DCs expressing sex-hormone receptors, whereas bone marrow or spleen-derived DCs are spared, which may possibly explain how the pregnant immune system tolerates a semi-allogenic fetus while protecting it from infections at the same time. The exact mechanism of this selective sparing remains unclear, but it reinforces the pleiotropic nature of DCs and their alluring ability to respond depending on the situation (44).

##### Monocytes/Macrophages/Neutrophils

Monocytes or macrophages, also important APCs, contribute to immune responses by phagocytosis and the production of cytokines. Decidual CD14+ monocytes secrete anti-inflammatory cytokines such as IL-10 and TGF-β and become tolerogenic under the influence of galectin-1 and macrophage inhibitory protein-1 (45). Uterine decidual macrophages also demonstrate prominent anti-inflammatory polarization during pregnancy, under the influence of Th2 cytokines (IL-4, IL-5, IL-10, IL-13) and high glucocorticoid concentrations, with converting from an inflammatory M1 phenotype to a non-inflammatory M2 phenotype (46). P4 further inhibits toll-like receptor (TLR)-4 mediated activation of macrophages, thereby suppressing innate immune response to prevent fetal rejection during normal pregnancy (47).

##### Mast Cells

The rising level of estrogen during pregnancy activates uterine mast cells (uMCs) *via* estradiol receptors, and they promote their degranulation to release histamine, which aids proper blastocyst implantation (by tissue remodeling) and placental development (48). The pro-secretory role of estrogen is further confirmed as specific ER antagonist tamoxifen inhibits MC degranulation both *in vitro* and *in vivo* (49). Elevated histamine levels also induce pregnant myometrial contractions *in-vivo*, and this may possibly explain the increased number of pre-term deliveries reported in females with systemic mastocytosis (50).

During pregnancy, the number of uMCs increases, and there is a shift from tryptase and chymase positive MCs (MC_TC_) to only tryptase positive (MC_T_) phenotype (48). These MC proteases (tryptase and chymase) activate matrix metalloproteinase (MMP)2 and MMP9 to mediate extracellular matrix degradation and facilitate delivery (51). Interestingly, the role of MCs in delivery is further corroborated by significant rise of pre-term deliveries in women with asthma, another MC-mediated disorder (52). Besides histamine and proteases, uMCs also release VEGF and galectin-1 (a glycan-binding protein), which support uterine neovascularization, fetal spinal artery (SA) remodeling; and placental development, fetal growth, respectively, (51, 53). uMCs collaborate with uNKs for SA remodeling, as evidenced by worsened SA remodeling in the simultaneous absence of both cell lines, compared to isolated deficiency (54). Recent evidence suggests that Mcpt5, secreted by uMCs and uNKs, is essential for proper SA remodeling in pregnant mice (55). Additionally, MCs secrete pro-inflammatory cytokines (IL-2, IL-12, TNF-α, and IFN-γ) in the early and late stages of pregnancy, and anti-inflammatory cytokines (IL-4, IL-10) during mid-pregnancy, to maintain the Th1 and Th2 dynamics during early/late and mid-pregnancy, respectively, necessary for a successful outcome (48). In addition to sex hormones, regulatory T-cells (Tregs) also promote IL-9 mediated proliferation of uMCs and angiogenesis at the murine feto-maternal interface to prevent early abortion (41, 56). Thus, there is a complex interplay between MCs, sex hormones, and immune cells during pregnancy, which may influence urticaria, as it is primarily a MC-mediated disorder.

##### Natural Killer Cells

NK cells, specifically the uterine variant (uNKs) constitute the major fraction of uterus lymphocytes in early pregnancy (~70%) and are responsible for maternal uterine vasculature remodeling and fetal survival (14). uNK cells differ from peripheral NK both structurally (differential expression of genes and receptor repertoire) and functionally (uNKs have lower cytotoxic activity compared to peripheral NKs) (14) Thus, the major function of uNKs is uterine vasculature remodeling and spinal artery formation, mediated primarily by the proangiogenic factor VEGF (57). Additionally, these cells secrete IFN-γ, a prominent anti-viral cytokine for fetal protection (58). However, there is confusion regarding the origin of uNK cells- whether they are recruited from peripheral NK cells into uterus, or they expand *in-situ* after pregnancy is established (14). Decidual NK cells increase in number under the influence of P4, IL-15, TGF-β, and stem-cell factor (SCF). P4 also promotes uNK cell recruitment in the pregnant uterus *via* secretion of osteopontin (59). Notably, a recent study has highlighted the role of decidual stromal cells in uNK proliferation in early pregnancy, by secreting IL-24, in an autocrine fashion (60).

##### Cytokines

Cytokines are polypeptides secreted by both innate and adaptive immune cells, which maintain a particular microenvironment, e.g., inflammatory or tolerogenic. The feto-maternal interface demonstrates a pro-inflammatory cytokine profile [IFN-γ, TNF-α, IL-1, IL-6, 1L-17, and the IL-6 family leukemia inhibitory factor (LIF)] during implantation and delivery, and an anti-inflammatory/tolerogenic profile (IL-10 and TGF-β) during the 2nd and 3rd trimester (61).

#### Changes in Adaptive Immunity

Adaptive immunity refers to the acquired and specific host defense system against previously exposed antigens, primarily involving T and B lymphocytes.

##### T Lymphocytes

Normal pregnancy reflects a pro-inflammatory Th1/Th17 profile at its early and late stages, essential for fetal implantation and onset of labor, respectively. Major adaptation occurs mid-gestation, involving a shift from the pro-inflammatoryTh1/Th17 spectrum toward Th2 immunity, thus creating a tolerogenic environment to ensure the survival of the semi-allogenic fetus (14, 23). E2 plays a major role in skewing immunity toward Th2 at the fetal-maternal interface along with depressing the inflammatory Th1 axis (20).

Although a conspicuous Th2 immunological shift occurs during pregnancy, absolute dominance of Th2 cytokines does not occur as evidenced by successful pregnancies in mice deficient in Th2 cytokines such as IL-4,5,9, and 13 (62). Recently, researchers have demonstrated up-regulation of soluble receptor antagonists of pro-inflammatory cytokines such as soluble IL-6 Ra, TNFRA and IL-1Ra, and expansion of Treg cells, in addition to Th2 cytokines, in healthy human pregnancies (63).

Apart from conventional T cells, CD4+ Treg cells are also involved in creating an anti-inflammatory milieu by “regulating/depressing” the immune system *via* cytokines like IL-10 and TGF-β and inhibition of decidual effector T-cells by silencing their chemokine genes. The concentration of Treg cells fluctuates during pregnancy and reaches its peak in mid-gestation, under the influence of P4, E2, and fetal antigens, to suppress the maternal immune system and prevent fetal rejection. The important contribution of Treg cells (CD4+CD25+) is further corroborated by worse pregnancy outcomes in their absence (64). A healthy pregnant uterus demonstrates increased endometrial expression of Foxp3, the major transcription factor of Treg cells, and its reduced expression has been associated with infertility (65). In addition to Treg cells, γδ T-cells (a minor fraction accounting for <5% of circulating T-lymphocytes) also increase in the feto-maternal interface and contribute to the local anti-inflammatory state by secreting IL-10, TGF-β, and PIBF (63).

##### B Lymphocytes

B lymphocytes are classically associated with antibody production; however, they also perform other roles such as antigen presentation and modulation of T-cell function. Notably, two types of antibodies (Abs) are produced- natural antibodies (autoreactive and cause autoimmune diseases- harmful for pregnancy) and asymmetric antibodies (AAbs) (needed for a successful pregnancy by reducing alloreactive responses). In normal pregnancy, natural Ab significantly reduces during the 3rd trimester to induce labor, while AAbs remain elevated during the entire pregnancy. Serum hCG regulates natural Ab production, while AAbs are controlled by P4 (66, 67).

Similar to Treg cells, Breg cells also increase during pregnancy, under the influence of hCG. These cells secrete IL-10 and inhibit Ab production by the B-cells, thus minimizing the chance of autoimmune disorders and graft (fetus) rejection (68).

#### Other Changes

Pregnancy-induced immunologic tolerance may increase maternal susceptibility toward various bacterial and viral infections. These infections might trigger inflammation and tissue destruction and stimulate auto-reactive T cells as a “bystander phenomenon”, and possibly worsen some autoimmune disorders (69).

Another interesting consequence of this altered immune status is the maternal gut microbiome remodeling, characterized by expansion of *Enterobacteriaceae* sp., which may facilitate metabolic and immunological adjustments for a successful pregnancy outcome (70). Although several authors have reported an association between chronic urticaria and gut microbial dysbiosis, studies are lacking in pregnant women (71, 72).

Recently, the concept of feto-maternal microchimerism has gained importance, which states that the maternal immune system acquires a state of immunological tolerance by means of transplacental feto-maternal cross-talk (transfer of genetically heterogeneous fetal material into maternal circulation) (63). Triche et al. (73) have shown HLA disparity between a mother and fetus is essential for a normal pregnancy, while feto-maternal HLA matching (class I and class II) has resulted in spontaneous abortion and pre-eclampsia.

**Figure 2:**
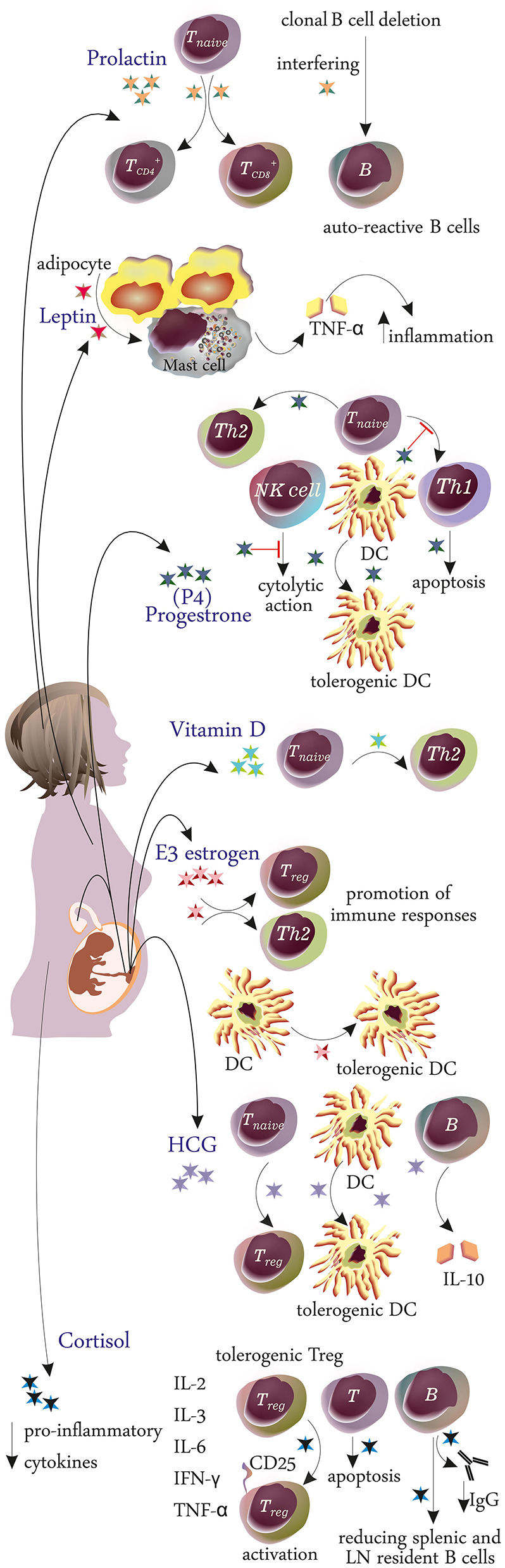
Effects of various hormonal changes on the immune system during pregnancy.

**Figure 3:**
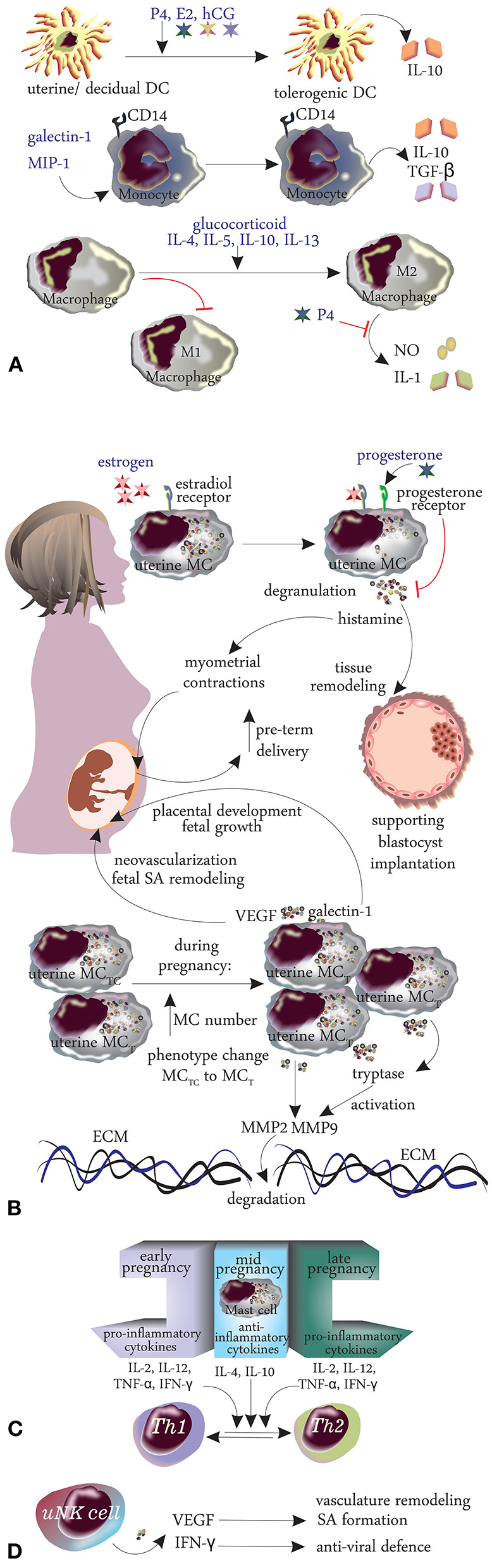
Changes in innate immunity during pregnancy. **(A)** Dendritic cells, monocytes, macrophages, mast cells. **(B)** Cytokines. **(C)** TH1/TH2 shift. **(D)** Natural killer cells.

The hormonal and immunological changes during pregnancy are summarized in Table 1 and Figures 2, 3.

**Table 1:** Hormonal and immunological changes during pregnancy.

**Name of hormone/type of immunity**	**Change during pregnancy**	**Role**
Progesterone (P4)	The maternal progesterone level continues to rise till about 10–12 weeks of gestation (corpus luteum), then returns to its baseline level and again starts to rise around the 32nd week of pregnancy (2nd peak-secreted by placenta) to be maintained until conception	P4 plays a crucial role in maintaining gestational tolerance by suppressing innate immunity and skewing the adaptive immunity toward the anti-inflammatory Th2 axis (20)
Estrogens	Estrogen concentration rises continuously until term, as it is primarily secreted by the fetoplacental unit	High concentration (as during pregnancy) favors the anti-inflammatory Th2/Treg responses (c.f. low concentration promotes the inflammatory Th1/Th17 pathway). It further depresses the innate immunity by programming DCs to become tolerogenic and anti-inflammatory (23)
Cortisol	The cortisol level maintains a steady rise during pregnancy and drops abruptly post-delivery	It exerts its anti-inflammatory effect by several modalities- reducing the levels of pro-inflammatory cytokines in circulation, activating tolerogenic Treg cells, inducing apoptosis of effector T-cells, and reducing the number of antibody-secreting B-cells (74)
Prolactin	Prolactin concentration slightly increases during gestation with the exponential rise during delivery and lactation period (c.f, sex hormones abruptly reduce after delivery). Secreted by the pituitary gland	It stimulates the immune system and causes its aberrant activation, and is associated with several autoimmune disorders (28)
Leptin	Its concentration rises to counter the hyper-metabolic state during pregnancy. Placenta is the 2nd source of leptin after adipose tissue	This hormone acts as a pro-inflammatory cytokine by activating the JAK-STAT, PI3K, and MAPK pathways (31)
Vitamin D	Placenta acts as the major extra-renal source of Vitamin D during pregnancy	Acts as an immunomodulator by promoting antibacterial innate immunity and suppressing inflammatory adaptive immunity (23)
HCG	Secreted by the placenta, its level peaks from 9th to 12th week, followed by a gradual decline until delivery	Its major role is stimulating the corpus luteum to secrete P4 (up to 10th/12th week). May have additional role in promoting maternal tolerance to ensure fetal survival (14)
AFP	Secreted by the yolk sac and fetal liver, this hormone peaks between 12 and 16 weeks, and gradually decline subsequently	Immunoregulatory role is currently under research (40)
Innate immunity	Uterine/decidual DCs secrete more IL-10 (anti-inflammatory cytokine)	Uterine DCs (professional APCs) become more tolerogenic and create a favorable local environment for the survival of the semi-allogenic fetus (14).
	Decidual CD14+ monocytes secrete more IL-10 and TGF-β. Macrophages change phenotypes from pro-inflammatory M1 to anti-inflammatory M2	A local (uterine) anti-inflammatory milieu is created, which facilitates the survival of fetus and prevents its rejection by hostile maternal immunity (46).
	Activation and degranulation of mast cells under influence of sex hormones	Histamine, released from MCs is necessary for fetal implantation and placental development. It may have a role in pregnancy CSU as histamine is its primary mediator (48). Additionally, VEGF secreted by uMCs aid in uterine vascular remodeling and fetal spinal artery development (14).
	Decidual NK cells increase in number and secrete VEGF (pro-angiogenic factor) and IFN-γ (anti-viral cytokine).	Plays an important role in the development of placenta, uterine vascular remodeling, and spinal artery formation. Additionally, protects the growing fetus from viral infections (14).
	Pro-inflammatory cytokines (IL-6 IL-1, TNF-α, IFN-γ, LIF) increase during 1st trimester and term, while anti-inflammatory cytokines (TGF-β, IL-10) predominate during 2nd and 3rd trimesters	Pro-inflammatory cytokines are necessary for fetal implantation and delivery, while anti-inflammatory cytokines (mid-gestation) create a tolerogenic local environment for the survival of semi-allogenic fetus (61)
Adaptive immunity	Pro-inflammatory Th1/Th17 profile (early and late stages) shifts toward anti-inflammatory Th2/treg axis (midgestation)	Early and late inflammatory milieu necessary for fetal implantation and labor, respectively. Midgestation anti-inflammatory tolerogenic profile ensures the survival of non-self fetus in the hostile maternal environment (14)
	Increase in CD4+ Treg cells and γδ T-cells at the feto-maternal interface, which peak during mid-gestation	These cells further suppress local adaptive immunity by secreting anti-inflammatory IL-10, TGF-β, and PIBF, to ensure fetal survival and prevent its rejection (63)
	Natural ABs (auto-reactive and harmful for pregnancy) significantly reduce during 3rd trimester, while asymmetric Abs (beneficial for pregnancy) remain elevated through pregnancy.	Drop in natural Abs induce labor and delivery, while asymmetric Abs protect the fetus by mitigating alloreactive responses (66)
	Regulatory B-cells (Breg) increase in number, under the influence of hCG	Secretion of anti-inflammatory IL-10 and reducing Ab production by B-cells, thus reducing the chance of fetal rejection and autoimmune disorders (68)

**Figure 4:**
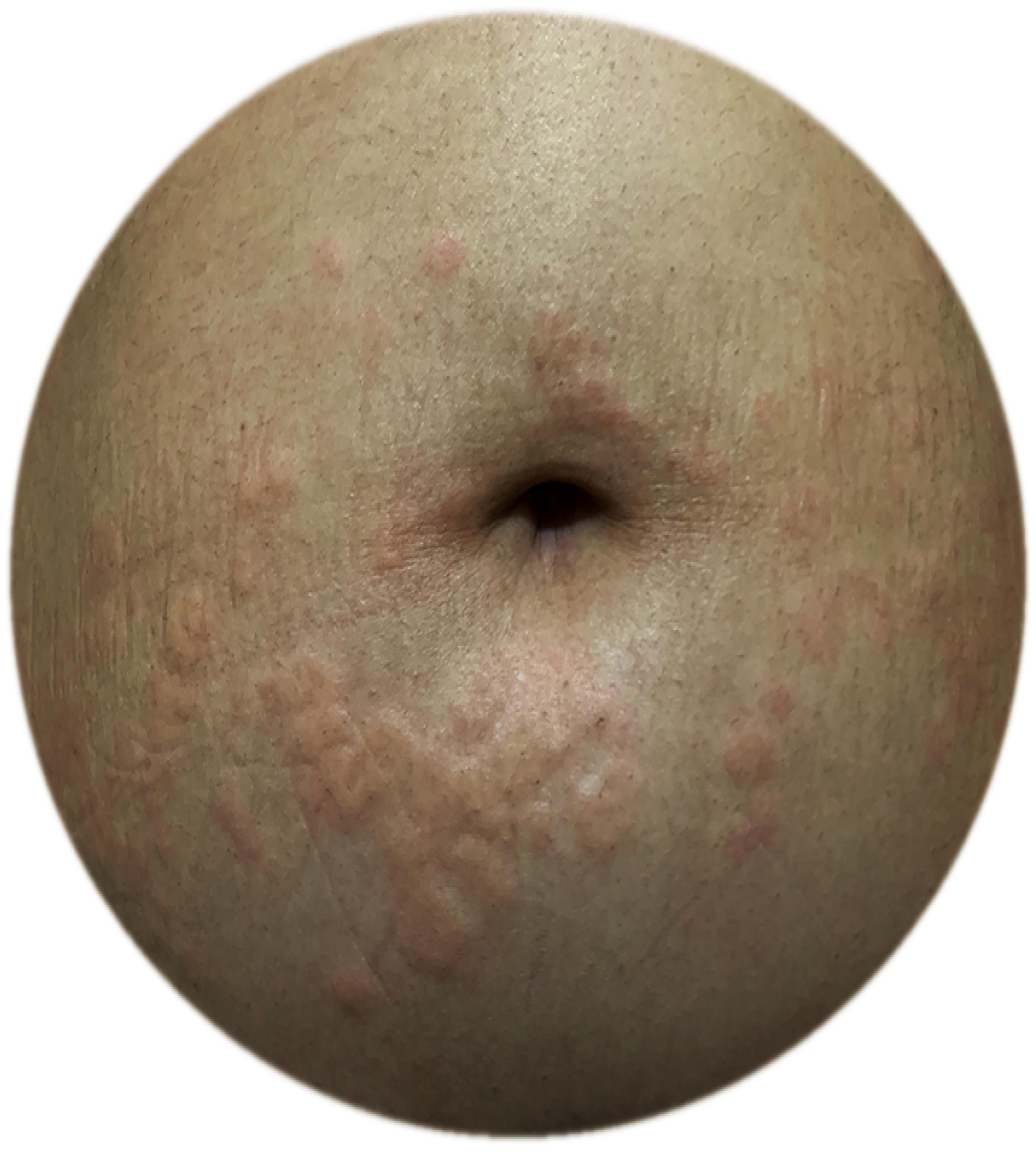
A pregnant CU patient with exacerbation of urticaria in the third trimester; urticarial plaques around the umbilicus.

## Disease Activity Changes During Pregnancy

During pregnancy, disease activity of chronic inflammatory disorders are subject to change due to the changes in immune responses such as decrease in Th1-type and Th17-type cytokines (that promote allograft rejection and may compromise pregnancy), increase in Th2-type cytokines (that inhibit the Th1 responses, promote allograft tolerance and therefore may improve pregnancy success) as well as an increase in T reg cells which dampen all the T helper responses and provides tolerance for fetal alloantigens and could induce fetoallograft tolerance through the production of IL-10 and TGF-β (75–77). As a result, Th2-type autoimmune disease get worsen and Th1/Th17-type autoimmune disease improve; i.e., rheumatoid arthritis (RA), multiple sclerosis, Graves' disease, and Hashimoto thyroiditis improve while systemic lupus erythematosus (SLE) and systemic sclerosis (SS) worsen during pregnancy (75). A favorable Treg–TH17 balance, the reduction in pro-inflammatory γδ T cells, and an increase in the soluble receptors that buffer the biological effects of TNF and IL-1 have been suggested to be the leading factors that contribute to pregnancy-related improvement of RA (63, 78, 79). Contrary to improvement in RA, lupus has been reported to deteriorate during pregnancy; however, the renal and skin lupus are differently affected by pregnancy; lupus nephritis deteriorates while skin lupus ameliorates during pregnancy. The worsened kidney function has been reported to be associated with renal inflammation and higher IFN-γ and IL-10 levels in the kidneys. IFN-γ by stimulating secretion of IgG antibodies and IL-10 by inducing B-cells to produce autoantibodies, which finally result in increased glomerular IgG deposition (80). In contrast, IL-10 was found to be increased and IFN-γ was decreased in the skin lesions of multiparous lupus-prone mice highlighting the role of IL-10 as a suppressor on skin lupus by possibly suppressing T-lymphocyte driven autoimmunity (81).

On the other hand, due to the increase in Th2 immune responses, an increased disease activity is anticipated in allergic disorders. That is, asthma exacerbations have been reported to range from 13 to 52% during pregnancy and most exacerbations occur in the second or beginning of the third trimester (82). Atopic dermatitis worsened during pregnancy in 52% in one study which also reported that most of the worsening occurred by 20 weeks of gestation (83) and in 61.0% in another (84).

Although CU is a very common and female-dominant disease that favors the reproductive age group, there is only one study that evaluates the effects of pregnancy on CU or the effects of CU on pregnancy outcomes, which is performed by the UCARE network (12, 85). In this study, 288 pregnant patients with CU from 21 centers/13 countries were asked to answer an a-47-item questionnaire which included questions on the exacerbations, angioedema attacks, emergency referrals, the overall course of CU during pregnancy, the course of urticaria after giving birth as well treatments before and during pregnancy, outcomes of pregnancy, and treatments given during breastfeeding. The study included both CSU and CIndU patients (CSU 66.9%, CIndU 12.8%, CSU + CIndU 20.3%) who experienced pregnancy within the last 3 years, and whose CU started before pregnancy (Figure 4). Disease activity before pregnancy was almost equally distributed among the patients (35.7% reported their disease activity as mild, 34.2% as moderate, and 29.7% as severe before pregnancy, respectively). Of 288 patients, 51% rated their CU as improved, 29% as worse, and 20% as unchanged during pregnancy. Two in five (43.5%) experienced acute CU exacerbations during pregnancy which most commonly occurred exclusively in the 3rd trimester (27.6%) or the 1st trimester (22.8%). Emergency referrals for CU were also most common in the 3rd trimester and angioedema occurrence was most common in the first trimester. The reason for the increase of disease activity during the first and third trimesters was explained by the predomination of Th1 immune responses and pro-inflammatory signals that promote MC activation in CU patients. These results were found similar to pregnant patients with mastocytosis who also showed exacerbation during the 1st or 3rd trimester (48).

The rate of emergency referrals (9.8%) and rate of angioedema (17.4%) during pregnancy were lower than the reported rates in non-pregnant CU patients (compared to 14.8 and 33.5%; and 40.3 and 45% in ASSURE and AWARE studies, respectively) (86, 87).

While the risk factors determined in the univariate analysis for CU worsening during pregnancy were having no angioedema and having mild disease activity before pregnancy, receiving no treatment before pregnancy, receiving treatment during pregnancy, having CIndU, worsening of CU during the previous pregnancy; after adjusting for cofounders, having mild disease before pregnancy and receiving treatment during pregnancy were left as the relevant risk factors for CU worsening during pregnancy.

After delivery, half of the patients (50%) whose urticaria improved during pregnancy reported worsening of CU after giving birth, while half (52%) with worsening of CU during pregnancy showed no change in their CU activity after giving birth. As an explanation to disease activity changes after birth, the authors proposed that the subsiding of Th2 skewing in the post-partum period results in the worsening of Th1 and Th17 autoimmune disorders and improvement in Th2-driven disorders (75) and concluded that CU patients with a dominant immune profile of Th1/Th17 might experience increased disease activity after birth, while CU patients with a Th2-linked autoallergic profile might show improvement.

As there were also patients who displayed disease activity increase during the second trimester and also patients whose urticaria had a worse course during pregnancy; the authors hypothesized that these patients might have the type 1 autoimmune (autoallergic) type of CSU.

## Management of Urticaria During Pregnancy

The management of CU depends on four major steps; (1) Disease activity assessment and monitoring (2) Education of patients (3) Control of triggering factors such as physical factors, NSAIDs and stress (4) Pharmacotherapy

Even though CU is a common disease in the reproductive female population, there is a lack of information on the safe use of recommended treatments and outcomes of pregnancy in pregnant CU patients. In the recent, already mentioned PREG-CU study, which evaluated the treatment patterns during pregnancy and lactation as well as the outcomes of pregnancy in 288 pregnant CU patients. The study evaluated the treatments before and during pregnancy, pregnancy trimesters which the treatments were received, outcomes of pregnancy, and treatments given during breastfeeding with a questionnaire. The results of the study showed that 81.4% of CU patients continued to use their medication when they decided to become pregnant. During pregnancy 60% of the patients used regular medication for CU with half of them (48.8%) did so during the whole pregnancy. During pregnancy, standard-dose sg-AHs (35%), standard-dose first-generation AHs (fg-AH) (7.6%), higher than standard-dose sg-AHs (5.6%), and omalizumab (5.6%) were the most commonly used treatments, respectively. Most commonly used AHs were cetirizine (37.4%), loratadine (14.6%); and levocetirizine and fexofenadine (7.3%; each). The outcomes of pregnancy in patients with CU were similar to the normal population: a preterm birth rate of 10.2% and newborn medical problems rate of 7.9%. No risk factors were found to be associated with preterm birth and newborn medical problems (88). Eight of 10 CU patients breastfed their babies and 54.3% of them used medication for CU while breastfeeding. Of them, 63.4, 14.1, and 6% used a standard-dosed sg-AH, higher than standard-dosed sg-AH and omalizumab; respectively.

The EAACI/WAO/EDF International guideline for the management of urticaria recommends to start treatment with standard doses of second-generation (non-sedative) H1 antihistamines (sg-AH), to increase the dose up to 4-folds in case of no response to standard doses of sg-AHs and if there is no response in 2–4 weeks to add on omalizumab as the third step (1). The recommended treatment in omalizumab-resistant cases is cyclosporine-A (1). The guideline recommends adopting the same approach in the management of pregnant and lactating patients but also emphasizes the lack of evidence-based information on the safety of urticaria treatments.

For getting information on the safe use of medications during pregnancy, we have been using the letter category system of the Food and Drug Administration (FDA). Based on data derived from human and animal studies, this system has classified the reproductive safety of medications in five risk categories (A, B, C, D, and X). However, in 2015 a new system called “Pregnancy and Lactation Labeling Rule” or PLLR is implemented given to the oversimplified or sometimes misleading nature of the pregnancy risk category system (89). The new PPLR format summarizes data on pregnancy, lactation, and exposure registries and includes a new section for men and women with reproductive potential. With this new system, physicians will be able to evaluate benefits vs. risks while counseling pregnant and nursing patients who need to take medication. This new system required that FDA drug submissions on or after 30th June 2015 be in the new PLLR format. The drugs which were approved between 30th June 2007 and 29th June 2015 should have transitioned to the new PLLR format by 30th June 2019 (90). However, it is not known if the foreseen FDA drug labels have been transitioned to the new PLLR format.

### H1 Antihistamines

The key elements of pharmacotherapy of CU are H1 antihistamines and antihistamines are among the most frequently prescribed medications during pregnancy. Approximately 15% of pregnant women use antihistamines during pregnancy, particularly during the first trimester (91). Despite associations of first- and second-generation H1AHs with birth defects have been reported in older reports, detailed analysis of the findings from these reports did not show a meaningful association between antihistamines and major congenital anomalies (92, 93).

Loratadine and cetirizine are the antihistamines of choice based on the data on their safety and the recommendations in the urticaria guidelines (1, 94–98). Compared with loratadine, the use of desloratadine during pregnancy did not increase the risk of adverse pregnancy outcomes (99) and a study from Denmark showed that use of fexofenadine during pregnancy did not increase the risk of major birth defects or spontaneous abortion compared with cetirizine (100).

The use of chlorpheniramine or diphenhydramine as first-generation H1 antihistamines is not recommended as the first-line treatment due to their various side effects. They have not been associated with adverse fetal outcomes in prospective cohort trials (97). The use of H1 antihistamines during the first trimester was not found to be associated with an increased risk of major malformations or other adverse pregnancy outcomes (91). Table 2 shows the pregnancy categories of H1-antihistamines. The safety of higher than approved doses of antihistamines has not been evaluated in pregnant patients, therefore potential risks and benefits have to be discussed with the patient before implementing it.

**Table 2:** Considerations for pregnancy and lactation for the medications used in the treatment of chronic urticarial.

**Medication**	**FDA pregnancy labeling and lactation rule**	**Pregnancy considerations**	**Lactation considerations**
Cetirizine	Pregnancy category B PLLR is available (https://pdf.hres.ca/dpd_pm/00035506.PDF)	May be used for the treatment of CU during pregnancy	Excretion in breast milk is considered low. High doses may cause drowsiness in infant
Loratadine	Pregnancy category B	May be used for the treatment of CU during pregnancy	Excretion in breast milk is considered low.
Chlorpheniramine	Pregnancy category B PLLR is available (https://www.accessdata.fda.gov/drugsatfda_docs/nda/2015/206323Orig1s000Lbl.pdf)	Not recommended by the guidelines; however, may be used for the treatment of CU during pregnancy if individually preferred	Occasional doses are acceptable. High doses might cause effects in infant or decrease the milk supply
Hydroxyzine	PLLR is available. In product monograph contraindicated in early (first trimester) pregnancy (http://eci2012.net/wp-content/uploads/2015/03/Atarax-En-Monograph-100902.04-Jan-2015.pdf)	Not recommended by the guidelines; however, may be used for the treatment of CU during pregnancy if individually preferred	Small doses may not cause any adverse effects in infants. High doses may cause drowsiness in infant or decrease the milk supply
Diphenhydramine	Pregnancy category B	Not recommended by the guidelines; however, may be used for the treatment of CU during pregnancy if individually preferred	Excretion in breast milk is considered low. May cause drowsiness in newborn
Montelukast	Pregnancy category B	Not recommended by the guidelines; however, may be used together with antihistamines if individually preferred	Excretion in breast milk is considered low
Omalizumab	Pregnancy category B PLLR is available (https://www.novartis.ca/sites/www.novartis.ca/files/xolair_scrip_e.pdf)	Recommended to use only in antihistamine refractory severe CSU cases after outweighing risks over benefits	Excretion in breast-milk is considered very low
Systemic corticosteroids	Pregnancy category B (for prednisolone & methyl-prednisolone) No adequate and well-controlled studies in pregnant women Should be used only if the potential benefit justifies the potential risk to the fetus	Recommended to use only for the treatment of CU exacerbations in the lowest effective dose for the shortest duration (use ≤ 20 mg/day)	Excretion in breast milk is considered very low
Cyclosporine	Pregnancy category C No adequate and well-controlled studies in pregnant women Should be used only if the potential benefit justifies the potential risk to the fetus	Avoidance recommended; only to be considered in very refractory CSU cases; requires monitoring for adverse events	Excretion in breast milk is considered low. Detectable in infant's blood

*PLLR, pregnancy and lactation labeling rule*.

### Montelukast

Although the use of leukotriene antagonists for the treatment of CU is not recommended by the international guidelines due to insufficient level of evidence (1), in case of intention to use during pregnancy, it will be useful to know that montelukast has been assigned pregnancy category B and no increase in major malformations were reported with the use of this medication during pregnancy (101).

### Omalizumab

Omalizumab is a recombinant IgG1 anti-IgE monoclonal antibody which is recommended in the treatment of antihistamine resistant CSU (1). Animal data on omalizumab (reproduction studies in cynomolgus monkeys) showed no maternal toxicity when administered throughout late gestation, delivery and nursing, subcutaneously in doses up to 75 mg/kg (12-fold the maximum clinical dose) as well as no impaired male or female fertility, embryotoxicity or teratogenicity and no adverse effects on fetal or neonatal growth (102). The Xolair Pregnancy Registry (EXPECT) which was designed to compare the maternal and neonatal outcomes of asthma patients treated with omalizumab (*n* = 250) or conventional drugs but not omalizumab (*n* = 1,153) during pregnancy. The prevalence of major congenital anomalies (8.1 vs. 8.9%), live births (99.1 vs. 99.3%), premature birth (15.0 vs. 11.3%) was similar between omalizumab treated and the conventional treatment groups (103). Given that this study includes only asthmatic patients and is an observational study, it is difficult to draw definitive conclusions for the safety of omalizumab in pregnant CU patients, however, there are several case reports on the safe use of omalizumab during pregnancy in CU patients (104).

Of note, omalizumab has a very long life of elimination half-life (26 days), and omalizumab exposure of the neonate would persist for weeks after birth. This may also translate to the exposure of the fetus to omalizumab which has been given to the patient even she was not aware of her pregnancy (of note: elimination of a given drug totally from the body takes 4–5 half-lives; in case of omalizumab this would take 26 × 5 = 130 days).

Another point to remember is that although all IgG subtypes can cross the placenta, IgG1 has the greatest transplacental transfer. It is expected that the lowest omalizumab exposure occurs during the first trimester of pregnancy and the greatest during the third trimester (105).

In 2019, European Medicine Agency updated the European Public Assessment Report and stated that omalizumab might be considered for use in pregnancy (82). In antihistamine refractory, severe CU patients, omalizumab may be a reasonable choice of treatment; however, the benefit-risk ratio should be reconsidered in every pregnant case individually and should be discussed with the patient in detail.

### Cyclosporine-A

Cyclosporine-A is the treatment recommended by the guidelines for CSU cases who do not respond to omalizumab treatment for 6 months. It is classified as category “C” in the FDA letter category system for pregnancy.

Bar Oz et al. reported in their meta-analysis which included 15 studies with 410 transplant patients that cyclosporine-A is not a major human teratogen but is associated with a trend toward increased risk of congenital malformations in the babies of transplant recipients and increased rates of prematurity (106).

Due to its side effects such as hypertension and nephrotoxicity which can potentiate gestational complications such as preeclampsia, cyclosporine-A is generally not recommended in pregnancy. It should be preserved for very severe cases only after other treatments have failed (107).

### Systemic Steroids

The use of systemic glucocorticosteroids (GCS) in CSU is limited only during exacerbations for short periods by the guidelines (1). GCS are generally not considered to be teratogenic but has been linked to growth retardation if fetal exposure (a median of 20 mg/day) happens during intrauterine development (108). Even though an increased risk of ~3-fold for oral clefts has been shown (109), the US National Birth Defects Prevention showed no increased risk of oral clefts with the 1st trimester use of GCS in a case-control study (110). It should be remembered that GCS use in pregnancy may lead to maternal side effects such as hypertension, gestational diabetes, and preeclampsia, and these can lead to poor pregnancy outcomes (i.e., intrauterine growth restriction, macrosomia, intrauterine fetal demise). Therefore, if possible, the use of GCS should be avoided in pregnancy, but if it must be used, it should be prescribed for severe cases at the lowest effective dose (≤ 20 mg/day prednisone) for a limited period (111, 112). Use of GCS during exacerbations of CU as short courses of 1–5 days with minimally effective dose is unlikely to cause pregnancy complications. With a short half-life and effective metabolization by 11-β-hydroxy-steroid present in the placenta, prednisone should be the steroid of choice (FDA category B). Fetal exposure is found ~10% of the maternal plasma level (113–115).

## Management of Urticaria During Lactation

### Antihistamines

Hence the transfer rate to breast milk is minimal, second-generation antihistamines are safe to use during lactation (116). Cetirizine, loratadine, and fexofenadine are the best studied antihistamines (117, 118). Higher doses of terfenadine and loratadine showed very minimal transmission to the milk (114, 119). In refractory cases of CU who are nursing their babies, higher doses of second-generation antihistamines might be safely used since the transfer rate to breast milk is minimal (114). First-generation antihistamines might lead to infant irritability and drowsiness (120) and are better not used during lactation.

### Systemic Steroids

GCS are considered to be safe to use during lactation by The American Academy of Pediatrics. They recommend the use of minimal effective dose for the possible shortest duration and to prefer prednisone or prednisolone over other GCS options (121). Since low amounts of prednisolone can transfer to breast milk, delaying breastfeeding for 4 h after maternal GCS ingestion to avoid plasma level peaks occurring 1 h after ingestion is recommended (122, 123).

### Montelukast

Montelukast is safe to use during breastfeeding given its very low levels in breastmilk. Since it is approved for use even in infants as young as 6 months of age, amounts ingested during breastfeeding by the infants are not expected to cause any adverse effects (124). A task force of respiratory experts reported that the use of these medications during breastfeeding is probably safe (125).

### Omalizumab

With a molecular weight of 145,058 Da, omalizumab is a large protein molecule and, likely, omalizumab transfer to the milk and, therefore the level in milk, is very low. It is partially destroyed in the gastrointestinal system of the infant and systemic absorption by the baby is probably minimal (126). Pregnant and nursing asthmatic patients have been followed in the EXPECT pregnancy registry for several years; 154 infants of these mothers were breastfed while their mothers were on omalizumab treatment. The results of this study showed that there is no difference in serious adverse events among the infants who received or did not receive omalizumab (102, 103). A case report of a CU patient who was treated with omalizumab during pregnancy and nursery showed that only 1/10,000 to 1/1,000 of omalizumab in the maternal serum is transferred into breast milk (127).

### Cyclosporine-A

Cyclosporine-A transfers to the milk <1% of the mother's weight-adjusted dosage. It does not cause adverse effects on infant's growth, development, or kidney function. However, if cyclosporine-A is used during lactation, infants should be monitored for the serum levels of cyclosporine-A to rule out toxicity (128).

The considerations for pregnancy and lactation for CU medications are shown in Table 2.

## Conclusion

Managing a pregnant patient with CU is often a challenge for treating physicians. Our review provides information on the hormonal and immunological changes across pregnancy and their potential relevance for CU, and we present what is known about the impact of pregnancy on CU. We also provide information on treatment options for pregnant patients with CU.

CU may improve, stay the same or worsen during pregnancy. This information as well as the fact that no treatment could end up with emergency referrals and worsening of the disease therefore requirement of more treatment should also be discussed with the patient.

For sure the ideal situation during pregnancy and lactation is “no pharmacologic therapy”, especially during the first trimester, however, it is almost impossible for a CU patient to have no disease activity during pregnancy. Therefore, treatment with the aim of zero or minimal disease activity with the least treatment should be commenced during pregnancy. The potential side effects of the medications should be balanced against the risks of inadequately treated disease for the mother and the fetus. These considerations should be discussed with the patient who is weighing the potential benefits of relief from the treated disease vs. the potential risks of the medication and an informed and shared decision should be made.

Currently, there is for sure, lack of sufficient information on the management of CU during pregnancy and questions remain to be answered are which treatments are safe to use during pregnancy, how CU manifests during pregnancy, which CU patients show amelioration or deterioration during pregnancy and are there biomarkers to show how CU will progress during pregnancy and lactation? To answer these questions, prospective studies with large patient populations which will determine patient characteristics both in the clinical level and in the molecular level are needed.

## Useful Links

ENTIS (European Network of Teratology Information Service) https://www.entis-org.eu/ UK Teratology information service (UKTIS) https://medicinesinpregnancy.org

German: https://www.embryotox.de/;French: http://www.lecrat.fr/; Dutch: https://www.lareb.nl/ Organization of Teratology Information Specialists https://mothertobaby.org/

For pregnancy registry studies for the relevant drug (https://www.fda.gov/science-research/womens-health-research/list-pregnancy-exposure-registries).

For lactation database visit the Drugs and Lactation Database (LactMed) [Internet]. https://www.ncbi.nlm.nih.gov/books/NBK501922/.

## Author Contributions

EK: concept. EK and MM: design. EK and IP: data collection or processing and writing. EK, IP, AZ, AK, DE-A-K, MC, and MM: analysis or interpretation and approval. EK, IP, AZ, and AK: literature search. DE-A-K: figures. All authors contributed to the article and approved the submitted version.

## Conflict of Interest

The authors declare that the research was conducted in the absence of any commercial or financial relationships that could be construed as a potential conflict of interest.

## Publisher's Note

All claims expressed in this article are solely those of the authors and do not necessarily represent those of their affiliated organizations, or those of the publisher, the editors and the reviewers. Any product that may be evaluated in this article, or claim that may be made by its manufacturer, is not guaranteed or endorsed by the publisher.

## Abbreviations

AAbs: Asymmetric antibodies
AFP: Alpha-fetoprotein
AH: Antihistamine
APCs: Antigen-presenting cells
ASST: Autologous serum skin test
Breg: Regulatory B-cells
CIndU: Chronic inducible urticaria
CSU: Chronic spontaneous urticaria
CU: Chronic urticaria
DCs: Dendritic cells
ERs: Estrogen receptors
FDA: Food and drug administration
Foxp3: Fork head box protein 3
GCs: Glucocorticosteroids
hCG: Human chorionic gonadotrophin
LIF: Leukemia inhibitory factor
MCs: Mast cells
NK cells: Natural killer cells
PIBF: Progesterone-induced blocking factor
PLLR: Pregnancy and lactation labeling rule
PRs: P4 receptors (progesterone receptors)
TLR: Toll-like receptor
TPO: Thyroid peroxidase
Tregs: Regulatory T-cells
uMCs: Uterine mast cells
VEGF: Vascular endothelial-derived growth factor
MMP: Matrix metalloproteinase.
